# Supplementary material for: Parkin-mediated mitophagy is negatively regulated by FOXO3A, which inhibits Plk3-mediated mitochondrial ROS generation in STZ diabetic stress-treated pancreatic β cells
Source: PLoS One. 2023 May 3;18(5):e0281496. doi: 10.1371/journal.pone.0281496 (PMC10155949; doi:10.1371/journal.pone.0281496)
Supplement: S1 File — (PDF) [file pone.0281496.s001.pdf]

Supplementary Figure S1

Gene ID	Clone ID	Species	Target sequence (5’- -3’)
PINK1	#1	Mouse	5’-GAACTTTGCTTGTCGTCAATA-3’
PINK1	#2	Mouse	5’-CCTGGCTGACTATCCTGATAT-3’
PINK1	#3	Mouse	5’-GTTCCTCGTTATGAAGAACTA-3’
PARK2	#1	Mouse	5’-GACCTGGAACAACAGAGTATT-3’
PARK2	#2	Mouse	5’-CGTTTCATTATCTGCAACTTTA-3’
PARK2	#3	Mouse	5’-CGGAGGATGTATGCACATGAA-3’
FOXO3A	#1	Mouse	5’-CCGGCACCAUGAAUCUGAAUGAUGG-3’
FOXO3A	#2	Mouse	5’-CAGUACCGUGUUUGGACCUUCGUCU-3’
FOXO3A	#3	Mouse	5’-CCAGUGACUUGGACCUGGACAUGUU-3’

Supplementary Figure S1. The target sequence of mouse *PINK1*, *PARK2* (Parkin), *FOXO3A*

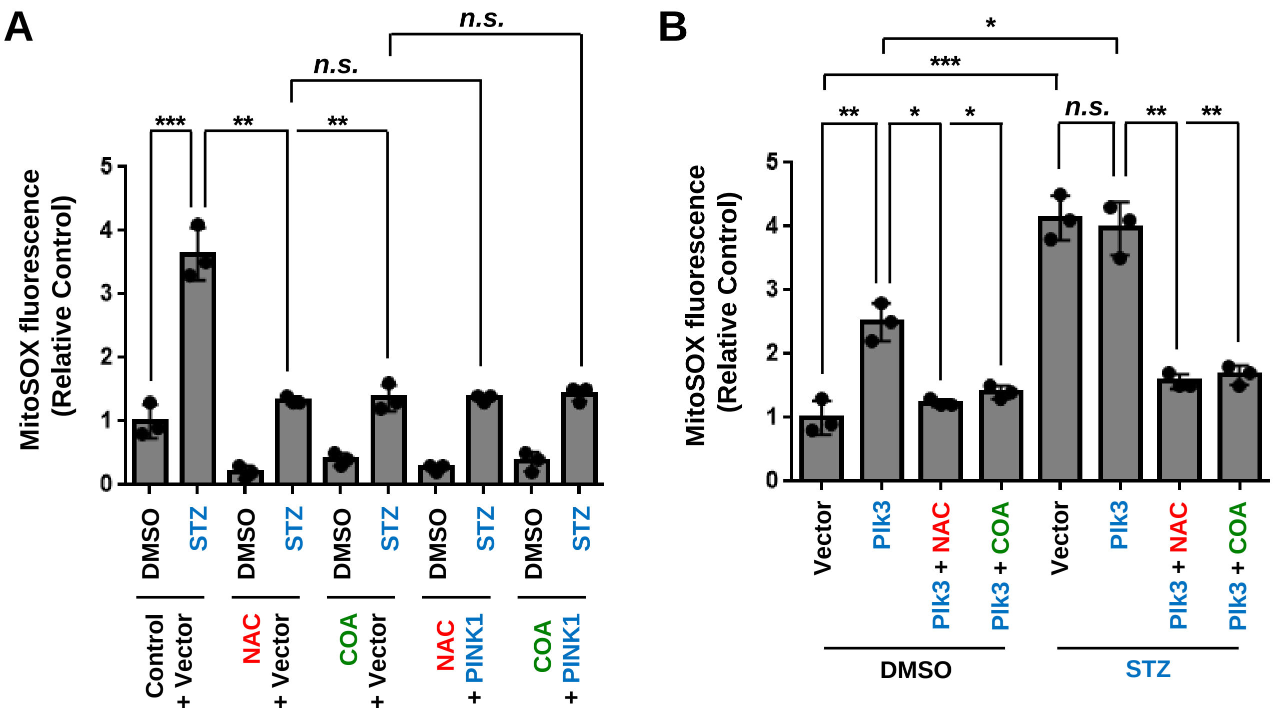

Supplementary Figure S2 Antioxidants block mitochondrial ROS generation by negatively regulating Plk3 increased by diabetic stress. (A,B) Beta TC-6 cells were treated with Streptozotocin for 12 h and then cells were transfected with the indicated plasmids or treated with antioxidants including NAC (10 mM) or natural COA water for 12 h. Cells were stained with MitoSOX (5 μM) for 30 min and then cells were analyzed by immunofluorescence assay and quantified. Representative images, scale bar, 20 μm. Statistical comparison of scatter plot and bar graph was performed by repeated measure ANOVA with multiple comparisons test; *$*P < 0.05$, $**P < 0.01$, $***P < 0.001$, n.s.*, non-specific. All experiments were repeated independently at least three times with similar results.

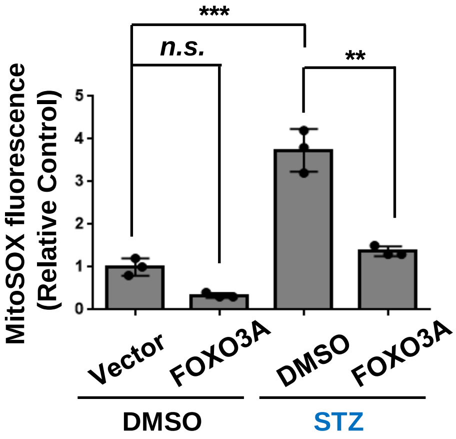

Supplementary Figure S3 overexpression of the FOXO3A gene significantly inhibited the increase of mitochondrial ROS induced by STZ stress. Beta TC-6 cells were transfected with the indicated plasmids and then cells were treated with Streptozotocin for 12 h. Cells were stained with MitoSOX (5 μM) for 30 min and then cells were analyzed by immunofluorescence assay and quantified. Statistical comparison of scatter plot and bar graph was performed by repeated measure ANOVA with multiple comparisons test; $^{**}P < 0.01$, $^{***}P < 0.001$, *n.s.*, non-specific. All experiments were repeated independently at least three times with similar results.
