# Supplementary material for: Parkin-mediated mitophagy is negatively regulated by FOXO3A, which inhibits Plk3-mediated mitochondrial ROS generation in STZ diabetic stress-treated pancreatic β cells
Source: PLoS One. 2023 May 3;18(5):e0281496. doi: 10.1371/journal.pone.0281496 (PMC10155949; doi:10.1371/journal.pone.0281496)

# Figure 1

## E

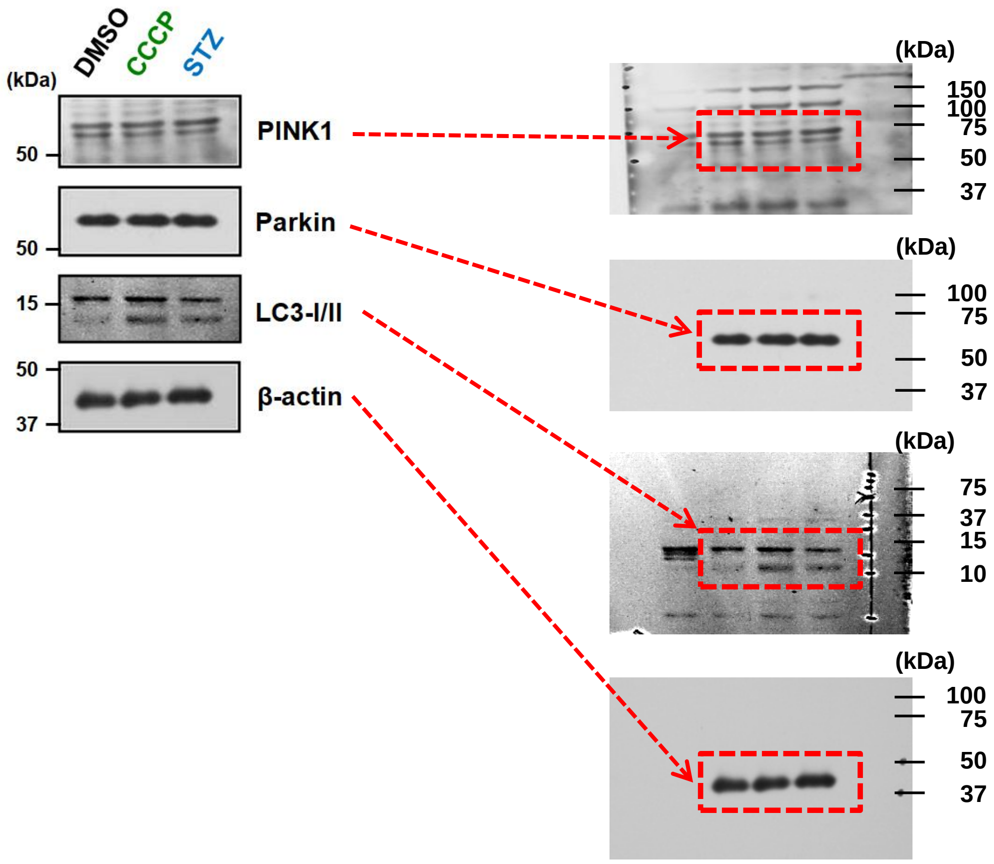


*(Uncropped scans continued on the next page)*

# Figure 1

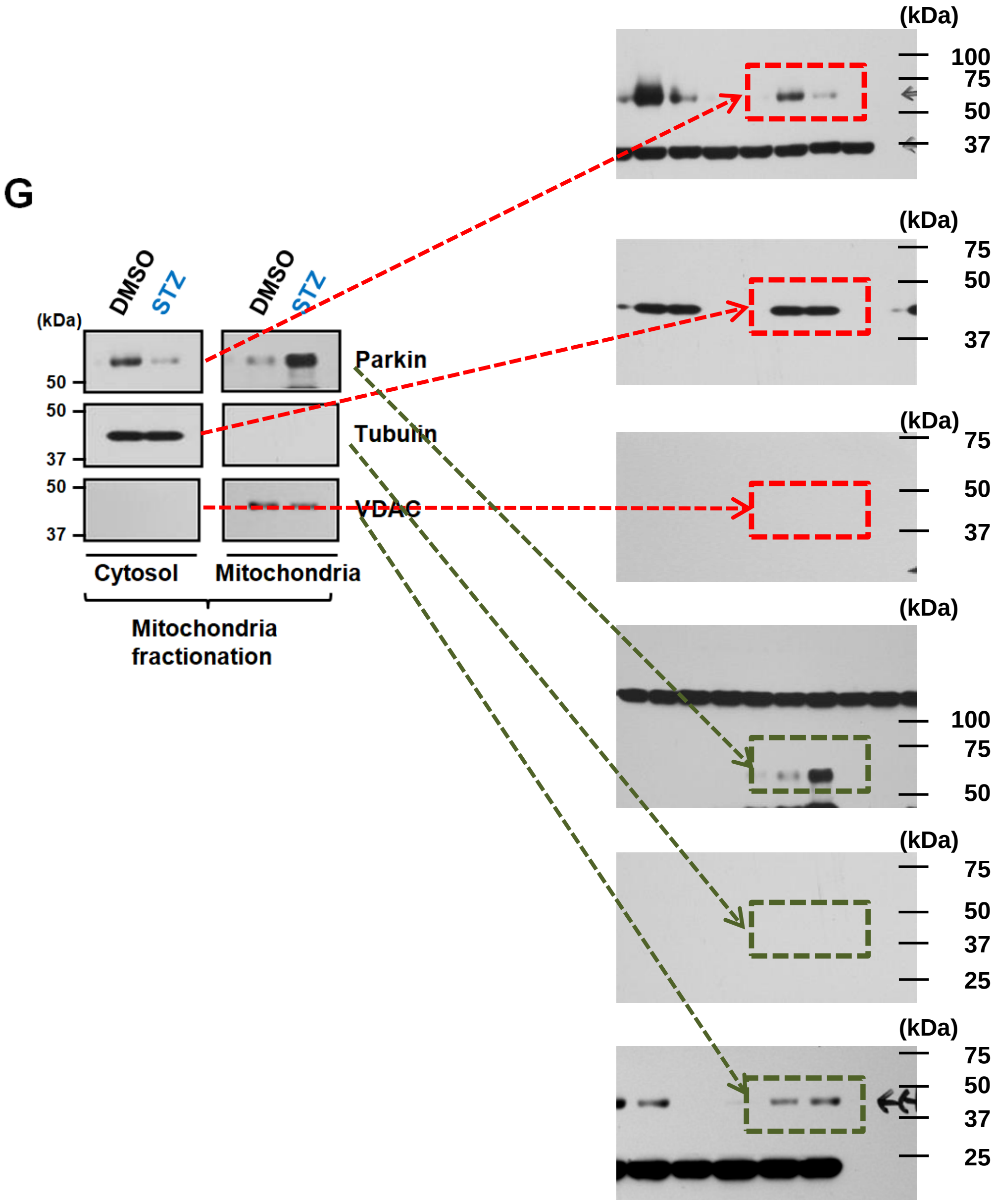


*(Uncropped scans continued on the next page)*

# Figure 2

A

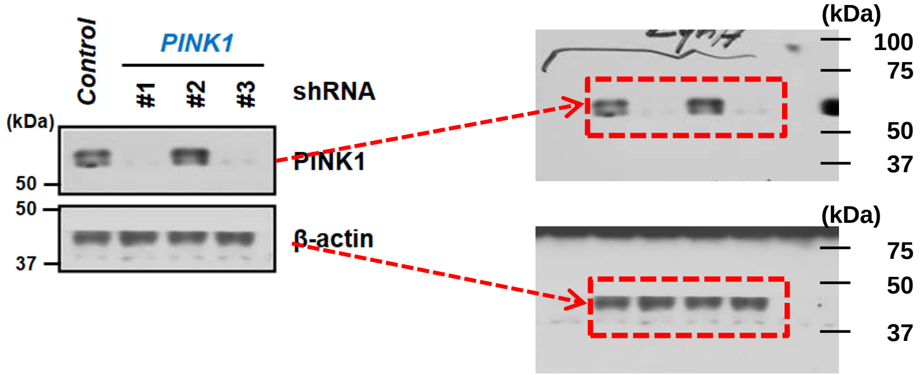


(Uncropped scans continued on the next page)

# Figure 2

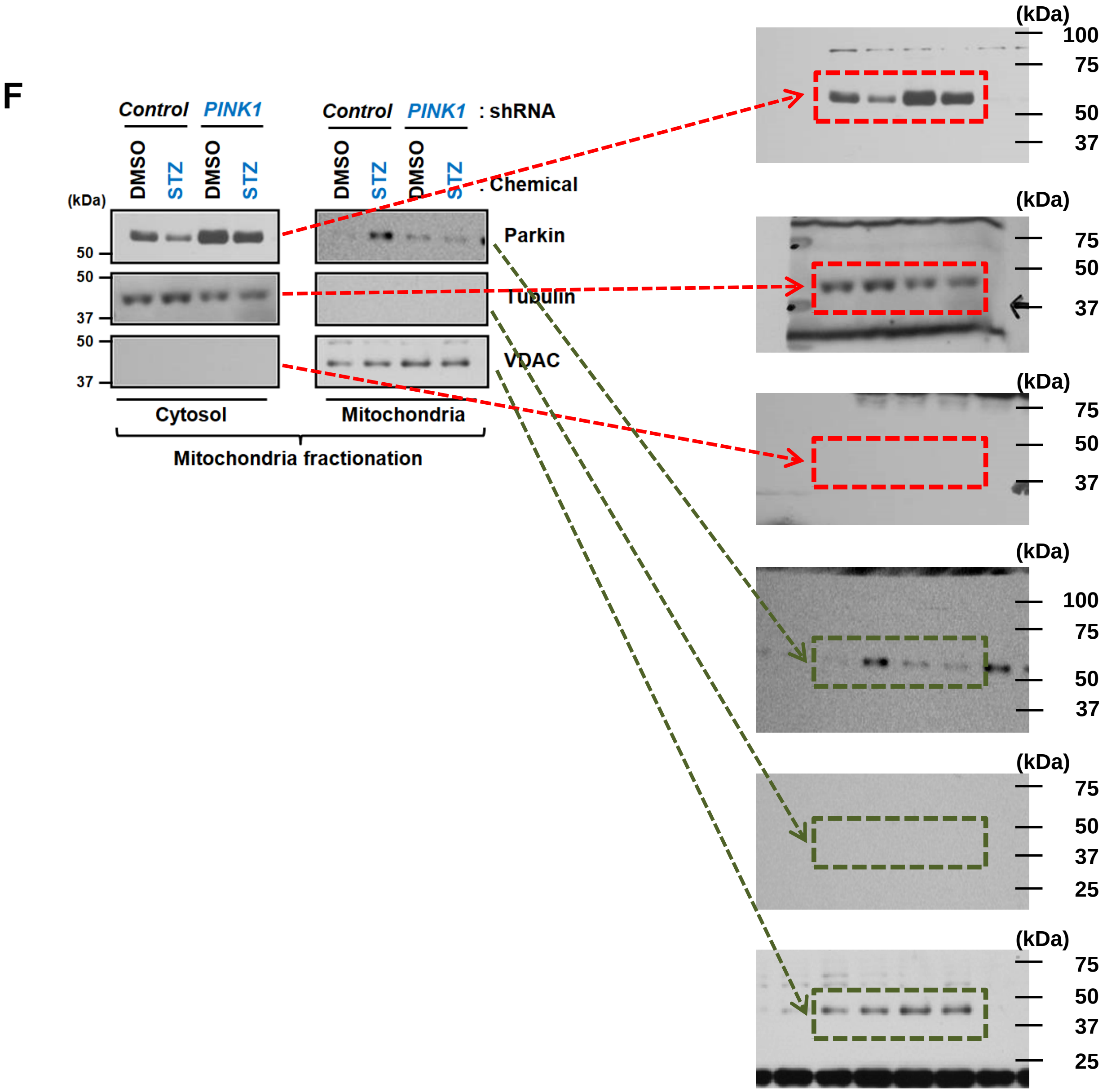
F
Control
PINK1
Control
PINK1
: shRNA
DMSO
STZ
DMSO
STZ
DMSO
STZ
DMSO
STZ
: Chemical
(kDa)
50
50
37
50
37
Parkin
Tubulin
VDAC
Cytosol
Mitochondria
Mitochondria fractionation
(kDa)
100
75
50
37
(kDa)
75
50
37
(kDa)
75
50
37
(kDa)
100
75
50
37
(kDa)
75
50
37
25
(kDa)
75
50
37
25

## Uncropped scans Figure 3

# Figure 3

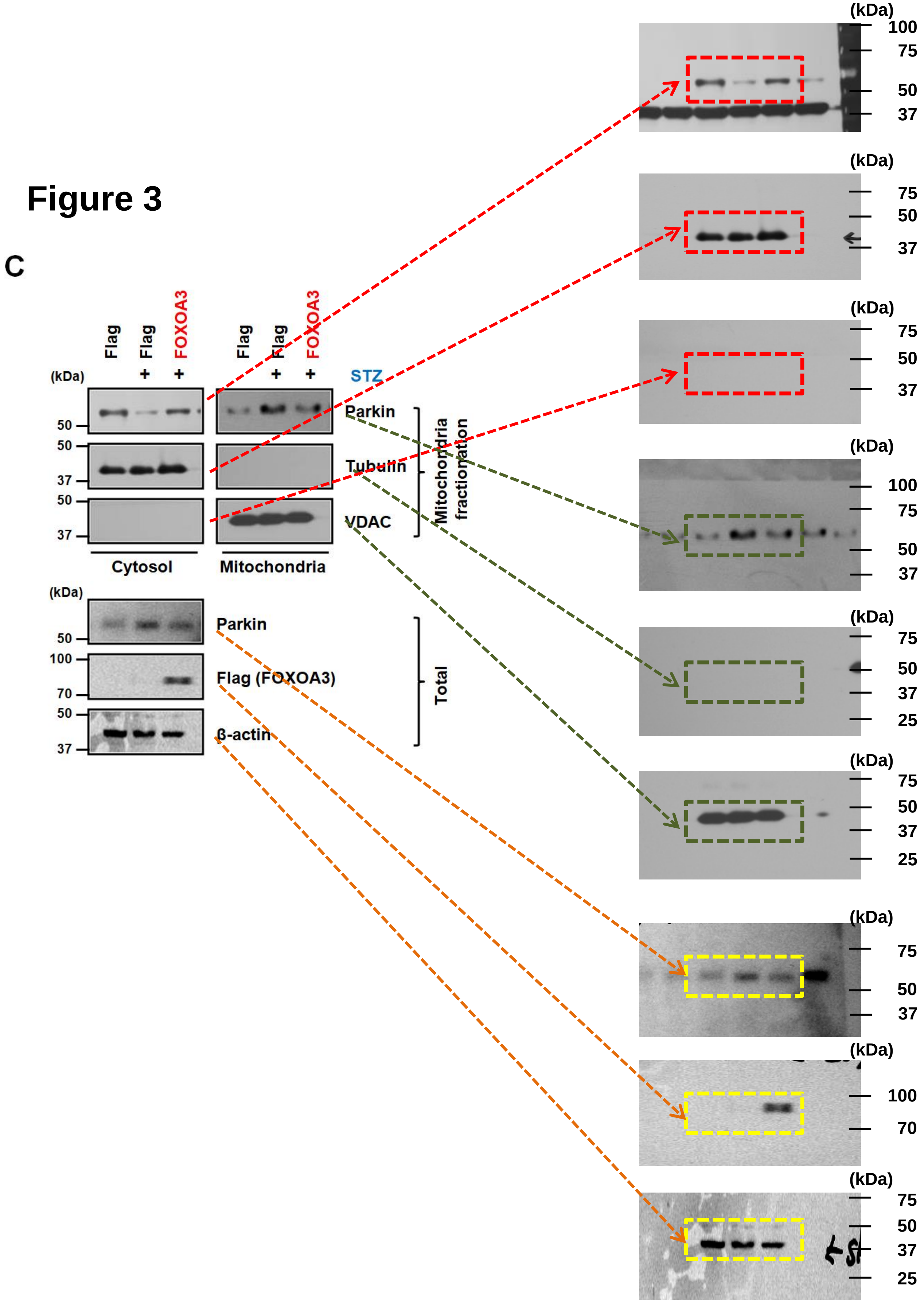

Supplement: S1 Raw images — (PDF) [file pone.0281496.s003.pdf]
